# Six-month efficacy of platelet-rich plasma for carpal tunnel syndrome: A prospective randomized, single-blind controlled trial

**DOI:** 10.1038/s41598-017-00224-6

**Published:** 2017-03-07

**Authors:** Yung-Tsan Wu, Tsung-Yen Ho, Yu-Ching Chou, Ming-Jen Ke, Tsung-Ying Li, Guo-Shu Huang, Liang-Cheng Chen

**Affiliations:** 1 Department of Physical Medicine and Rehabilitation, Tri-Service General Hospital, School of Medicine, National Defense Medical Center, No. 325, Sec. 2, Cheng-Kung Road, Neihu District, Taipei, Taiwan Republic of China; 2 Integrated Pain Management Center, Tri-Service General Hospital, School of Medicine, National Defense Medical Center, No. 325, Sec. 2, Cheng-Kung Road, Neihu District, Taipei, Taiwan Republic of China; 3 School of Public Health, National Defense Medical Center, No. 161, Sec. 6, Minquan East Road, Neihu District, Taipei, Taiwan Republic of China; 4 Department of Radiology, Tri-Service General Hospital, School of Medicine, National Defense Medical Center, No. 325, Sec. 2, Cheng-Kung Road, Neihu District, Taipei, Taiwan Republic of China

## Abstract

Recently, a few small reports with short follow-up period have shown clinical benefits of platelet-rich plasma (PRP) for peripheral neuropathy including one pilot study and one small, non-randomized trial in patients with carpal tunnel syndrome (CTS). Therefore, we conducted a randomized, single-blind, controlled trial to assess the 6-month effect of PRP in patients with CTS. Sixty patients with unilateral mild-to-moderate CTS were randomized into two groups of 30, namely the PRP and control groups. In the PRP group, patients were injected with one dose of 3 mL of PRP using ultrasound guidance and the control group received a night splint through the study period. The primary outcome measure was the visual analog scale (VAS) and secondary outcome measures included the Boston Carpal Tunnel Syndrome Questionnaire (BCTQ) score, the cross-sectional area (CSA) of the median nerve (MN), electrophysiological findings of the MN, and finger pinch strength. The evaluation was performed before treatment and at 1, 3, and 6 months post-injection. The PRP group exhibited a significant reduction in the VAS score, BCTQ score, and CSA of MN compared to the those of control group 6 months post-treatment (p < 0.05). Our study demonstrates that PRP is a safe modality that effectively relieves pain and improves disability in the patients with CTS.

## Introduction

Carpal tunnel syndrome (CTS) is the most common peripheral entrapment neuropathy. The gradual ischemia resulting from high pressure with accompanying compression of the median nerve (MN) within the carpal tunnel is thought to contribute to the pathophysiology of CTS^1^. Moreover, CTS can cause inflammation of the intracarpal tendon including the flexor pollicis longus, deep and superficial flexor tendons. The inflammation of the tendon frequently produces a cycle of intracarpal swelling causing further compression of MN^2^. Typical symptoms and signs include numbness, tingling, pain, or burning sensation in the digits controlled by the MN, and/or nocturnal paresthesia. Thenar muscle wasting might also occur during the chronic stages^1^.

Treatments for CTS range from conservative strategies (medication, night splint, steroid injections, and physical therapy) to surgical decompression of the MN. Despite the availability of conservative therapies, their efficacy is usually unfavorable or short-lived^3^. A report revealed that approximately 60 to 70% patients with CTS who underwent conservative treatment still had symptoms after 18 months’ follow-up^4^. Moreover, a recent study has shown that the treatment failure rate of the wrist splint was reported as 69% after 12 months’ follow-up^5^. Although surgical intervention is more effective than conservative treatment, conservative therapies are advocated for mild-to-moderate CTS. Surgical therapy is suggested for severe CTS or patients with poor response to conservative treatments, since the failure rate of surgery ranges from 7–75%^6, 7^. Therefore, it is important to explore and develop a novel non-surgical intervention for CTS.

Platelet-rich plasma (PRP) is a biologic product of concentrated platelets and contains several growth factors that promote wound healing/growth, angiogenesis, and axon regeneration. PRP has been widely used as a safe and novel treatment in dentistry, orthopedics, ophthalmology, neurosurgery, and cosmetic surgery for three decades^8^. Recently, increasing evidence has revealed the beneficial effects of PRP on axon regeneration and neurological recovery in animal or *vitro* studies^9–16^. Since 2014, a few studies have applied PRP for treating clinical peripheral neuropathy, with acceptable success rates^17–21^. Among these studies, there was one pilot study and one small, non-randomized trial enrolling patients with CTS. It would be helpful to examine whether PRP aids in the promotion of extensive axon regeneration in the human population. However, the definite clinical effects of PRP on peripheral neuropathy are unclear due to small population sizes and short follow-up periods in published reports. This points to the need for a well-designed study to evaluate the effects of PRP on clinical regeneration following peripheral nerve injury.

In the present study, we investigated the 6-month effects of PRP in patients with mild-to-moderate CTS.

## Methods

### Study design

This study was designed according to the CONSORT 2010 statement^22^. This prospective, randomized, single-blinded, controlled study was conducted at Tri-Service General Hospital, Taiwan from November 2015 to October 2016. The study protocol was reviewed and approved by the Institutional Review Board of Tri-Service General Hospital (No. 1-104-05-108). All enrolled patients provided written, informed consent for the study. The study was performed in accordance with the principles of Declaration of Helsinki. The study was registered at www.ClinicalTrials.gov (number NCT02539186) on 8/31/2015. All methods for each subject were performed in accordance with the approved ethical guideline and there were no changes made to this trial after the commencement of the recruitment.

Eighty patients diagnosed with mild-to-moderate unilateral CTS were assessed for eligibility, and 60 were enrolled. The enrolled patients were block randomized in a 1:1 ratio into two groups, control and PRP groups, by an independent researcher via computer-generated randomization of study numbers (Microsoft Excel, Microsoft Inc., Redmond, WA, USA). The PRP group received one dose of ultrasound-guided PRP injection, while the control group wore a wrist splint. The splint was applied in a neutral position to restrict the wrist as previously described^3, 23^. The controls were instructed to put on the splint overnight for at least 8 hours daily throughout the study period. All participants were instructed to refrain from any other management approaches for symptoms resulting from CTS such as analgesics, steroid injections, or physical therapy, from 2 weeks before and throughout the study period, and were requested to report receiving any of these therapies.

### Inclusion and exclusion criteria

Participants diagnosed with mild-to-moderate unilateral CTS with clinical symptoms for at least 3 months undergoing electrophysiological study and ultrasonography were enrolled. The clinical symptoms and signs for the diagnosis of CTS were summarized in Table 1. If the patients met criterion 1 and one or more of criteria 2–4, the clinical diagnosis of CTS was established^24, 25^.

**Table 1:** Summarization of inclusion and exclusion criteria.

Inclusion criteria of symptoms and signs
1. Paresthesia/dysesthesia, painful swelling with clumsy weakness of the hand exacerbated by sleep or repetitive use of the wrist, and relieved by shaking the hand with postural change.
2. Sensory loss with numbness in the median nerve-innervated regions of the hand.
3. Weakness with atrophy of the median nerve-innervated thenar muscles.
4. Positive Phalen’s test and/or Tinel’s sign.
**Exclusion criteria**
1. History of wrist surgery, polyneuropathy, brachial plexopathy, or thoracic outlet syndrome.
2. History of thrombocytopenia, platelet dysfunction, systematic infection, pregnancy, and rheumatologic disorders.
3. Previous steroid injection for carpal tunnel syndrome.

The cut-off points or normal range of the electrophysiological study for CTS in this study were as follows: (1) upper limit of the median sensory nerve distal latency is ≤3.6 ms at a distance 14 cm away from the active recording; (2) difference in distal latencies between the ulnar and median sensory nerve is <0.4 ms; and (3) upper limit of distal motor latency (DML) of the MN is <4.3 ms at a distance 8 cm away from the thenar muscle belly^26–28^. Patients were excluded if they met one of the exclusion criteria (Table 1).

### Grades of CTS

Patients with mild and moderate CTS were categorized by the electrophysiological classification of CTS by Padua *et al*.^28^: mild: only abnormal digit/wrist sensory nerve conduction velocity (SNCV) with normal DML; moderate: abnormal digit/wrist SNCV and abnormal DML; or severe: absence of SNCV and abnormal DML.

### PRP preparation

Ten milliliters of blood sample were drawn from the antercubital vein using RegentKit-THT-1 (RegenLab SA, Mont-sur-Lausanne, Switzerland) followed by centrifugation at 3400 rpm for 15 minutes at room temperature using Regen Lab PRP Centri, yielding 3.5 mL of PRP^29^. The RegentKit-THT-1 has sodium citrate solution as an anticoagulant, and autologous thrombin as an activator to advance platelet activation and conversion of fibrinogen to fibrin. For quality tests, 0.5 mL of the PRP sample was sent to the laboratory and 3 mL was used for the ultrasound-guided injection. The concentration of platelets and leukocytes in the PRP was approximately 2.7 ± 0.4 times and 1.2 ± 0.4 times compared to that in whole blood, respectively.

### Ultrasound-guided PRP injection

**Figure 1:**
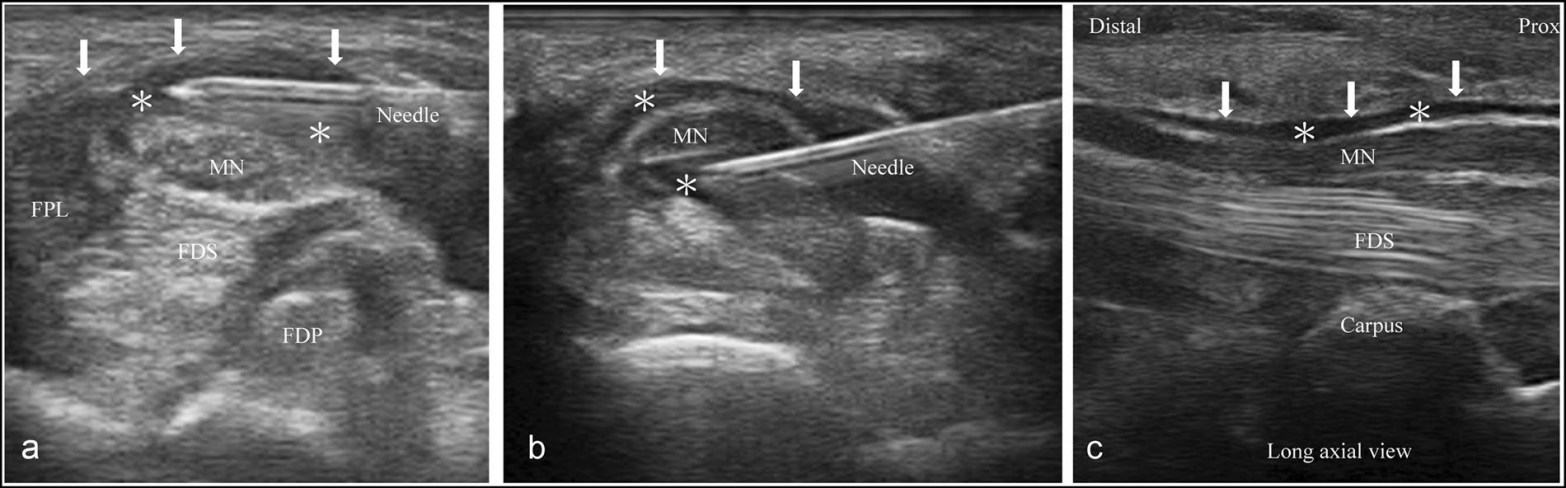
Ultrasonographic imaging. (**a**) Transverse view showing the median nerve (MN) peeled off from the flexor retinaculum (arrows) using 2 mL of PRP via hydrodissection (asterisk). (**b**) Transverse view showing the MN separated from the inferior underlying subsynovial connective tissue using an additional 1 mL of PRP via hydrodissection (asterisk). (**c**) Long axial view showing the distribution of PRP throughout the proximal-to-distal area of the carpal tunnel (asterisk). MN: median nerve; FPL: flexor pollicis longus; FDS: flexor digitorum superficialis; FDP: flexor digitorum profundus.

The ultrasound-guided PRP injection was performed by the same physiatrist (Dr. Wu), using ultrasonography (MyLab™ 25Gold, Esaote, Genova, Italy). With the palm facing upwards and the wrist slightly extended, the MN was identified at the inlet of the proximal carpal tunnel (pisiform level)^30, 31^. The ultrasound-guided injection was conducted using the in-plane ulnar approach^32^. The ulnar artery was identified using Doppler imaging, and a 25-gauge needle was passed from the ulnar side of the wrist toward the MN. After placing the needle tip on the MN, 2 mL of PRP was injected to peel the nerve off the flexor retinaculum via hydrodissection (Fig. 1a). An additional 1 mL of PRP was delivered to the inferior part of the MN and the MN was peeled from the underlying subsynovial connective tissue (Fig. 1b). After this, the entire carpal tunnel was scanned to ensure that the PRP had spread throughout the proximal-to-distal area of the carpal tunnel (Fig. 1c). All patients were observed for 10 minutes after injection for possible dysesthesia or bleeding.

### Outcome measurements

One physiatrist (Dr. Ke) with 5 years’ experience in musculoskeletal ultrasonography and electrophysiological study, who was blinded to the patients’ randomization, performed all the measurements in all patients of both groups before intervention and at months 1, 3, and 6 after treatment.

### Primary outcomes

#### Visual analog scale (VAS)

Digital pain severity and paresthesia within 7 days of the assessment were measured using the VAS, with 10 points indicating extremely severe pain and 0 points indicating no pain^33^.

### Secondary outcomes

#### 1. Boston Carpal Tunnel Syndrome Questionnaire (BCTQ)

BCTQ was employed to evaluate the severity of symptoms and functional status with reproducibility, internal consistency, and validity for patients with CTS^34^. The subscale of symptom severity consists of 11 questions and the scores range from 1–5 points. The functional status subscale has 8 questions, and scores range from 1–5 points; higher scores mean worse severity and dysfunction.

#### 2. Cross-sectional area (CSA) of MN

CSA of the MN was evaluated as previously described^23, 30^. In brief, CSA was measured with an electronic caliper at the proximal inlet of the carpal tunnel using the pisiform bone as a landmark. The average of CSA was calculated using three measurements each.

#### 3. Electrophysiological study

The antidromic SNCV and DML of the MN were measured in all patients by using SierraWave, Cadwell (USA)^35^. The antidromic SNCV was examined as described in our previous study^23^. The DML was recorded via MN stimulation 8 cm proximal to the active electrode over the abductor pollicis brevis muscle. The average of SNCV and DML were calculated using three measurements each.

#### 4. Finger pinch (FP)

The palmar FP force was detected with a Jamar dynamometer (Fabrication Enterprises Inc., USA) as described in our previous study^23, 36^. The average of three test values was used for statistical analysis.

### Sample size

To reduce type II errors and increase the power, a preliminary power analysis using G* power 3.1.9.2 (UCLA, Los Angeles, CA, USA) [power (1-β) = 0.9; α = 0.05; effect size = 0.45] indicated that a sample of 54 people was required^37^.

### Data analysis

Statistical analyses were conducted with IBM SPSS Statistics Version 22. Demographic statistics were analyzed using the independent t-test for continuous data and X^2^ test for categorical data. The repeated-measures ANOVA followed by post hoc tests was performed for the data at various follow-ups. The independent t-test was used to compare the differences between the groups. All statistical tests were two-tailed and a p-value less than 0.05 was considered as statistically significant.

## Results

**Figure 2:**
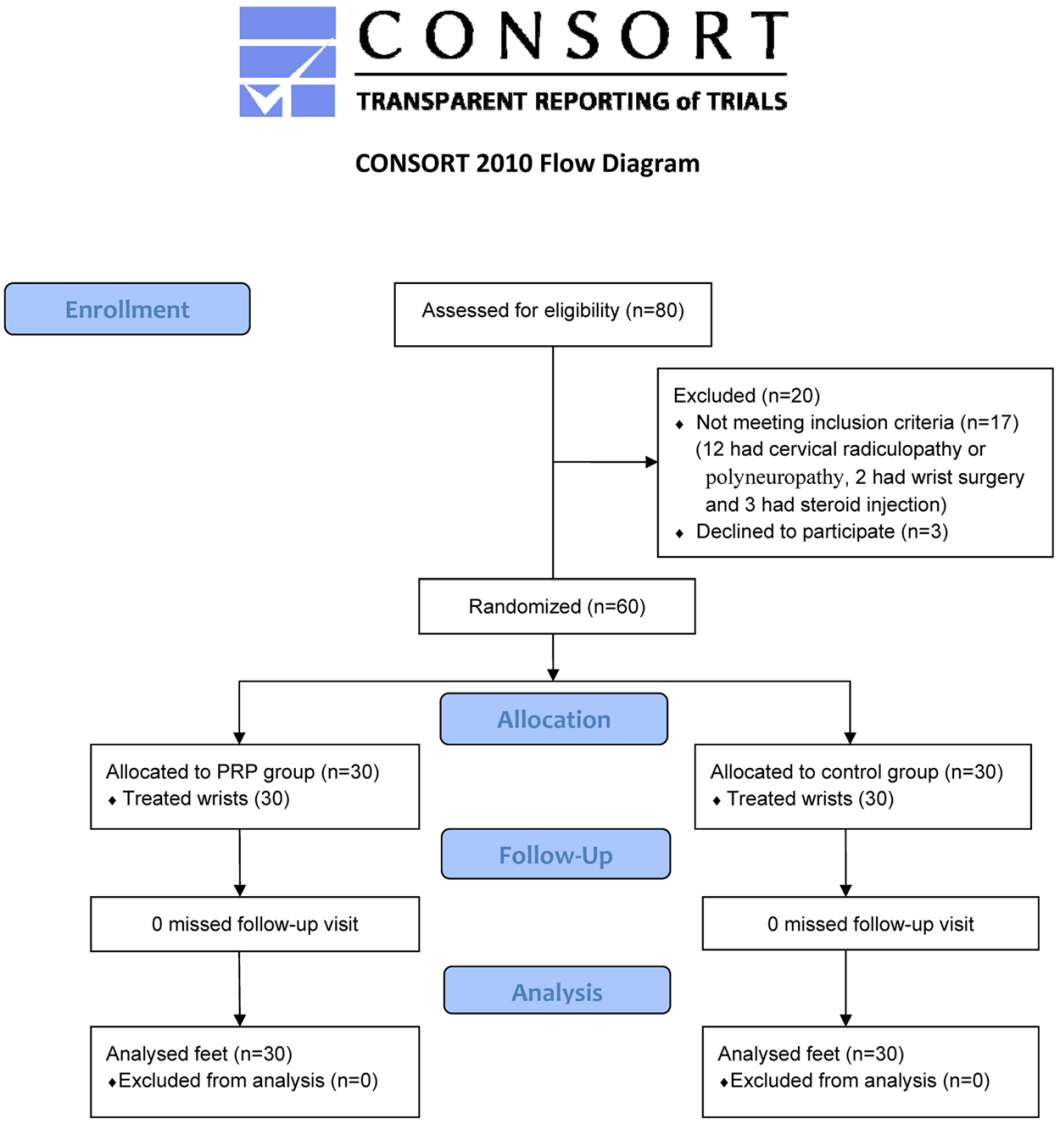
Study flow diagram^22^.

All 60 participants completed the study and 30 wrists in each study group were analyzed (Fig. 2). The clinical characteristics of participants are summarized in Table 2, and there were no significant differences in the variables between two groups. More than 80% of the patients were graded as having moderate CTS. Table 3 shows the VAS scores, BCTQ scores, electrophysiological study, CSA of the MN, and FP before and after treatment. Comparing the baseline data, a significant improvement in all outcome measures was observed in the PRP and control groups at all follow-up assessments (p < 0.05) (not including the 1^st^ month FP in the control group, p = 0.071).

**Table 2:** Baseline demographic and clinical characteristics of study participants.

	PRP group (n = 30)	Control group (n = 30)	*p* value
Age (year) (SE)	57.87 ± 1.51	54.27 ± 1.34	0.08
Body height (cm)	154.80 ± 0.63	156.57 ± 1.19	0.197
Body weight (kg)	62.23 ± 1.28	62.13 ± 2.33	0.97
Diabetes mellitus (n)	4 (13.33)	3 (10.00)	1.000
Hypertension (n)	9 (30.00)	11 (36.67)	0.784
Sex			0.706
Male (n) (%)	3 (10.00)	5 (16.67)	
Female (n) (%)	27 (90.00)	25 (83.33)	
Duration (months) (SE)	34.43 ± 5.67	30.70 ± 6.03	0.654
Dominant hand			—
Right (n) (%)	30 (100%)	30 (100%)	
Left (n) (%)	0 (0)	0 (0)	
Lesion site			0.796
Right (n) (%)	15 (50.00)	17 (56.67)	
Left (n) (%)	15 (50.00)	13 (43.33)	
Grading (Padua)			1.000
Moderate	25 (83.33)	26 (86.67)	
Mild	5 (16.67)	4 (13.33)	
VAS	6.50 ± 0.30	6.29 ± 0.31	0.631
BCTQs	26.17 ± 1.10	24.93 ± 1.22	0.457
BCTQf	19.23 ± 1.08	18.13 ± 0.65	0.387
FP (kg)	3.27 ± 0.28	3.74 ± 0.11	0.133
SNCV (m/s)	30.18 ± 1.29	32.35 ± 1.10	0.205
DML (ms)	5.66 ± 0.27	5.21 ± 0.23	0.215
CSA (mm^2^)	14.01 ± 0.82	12.91 ± 0.81	0.343

SE = Standard error; VAS = Visual analog scale; BCTQ = Boston Carpal Tunnel Syndrome Questionnaire (s = severity and f = function); FP = Finger pinch; SNCV = Sensory nerve conduction velocity; DML = Distal motor latency; CSA = Cross-sectional area.

**Table 3:** All the Outcome variables in each group before and after treatment.

	PRP group (n = 30)		Control group (n = 30)	
	Mean ± SE	*p* value	Mean ± SE	*p* value
VAS-Pre	6.50 ± 0.30		6.29 ± 0.31	
VAS month 1	3.89 ± 0.28	<0.001	3.88 ± 0.28	<0.001
VAS month 3	2.91 ± 0.23	<0.001	3.36 ± 0.26	<0.001
VAS month 6	1.97 ± 0.23	<0.001	2.99 ± 0.27	<0.001
BCTQs-Pre	26.17 ± 1.10		24.93 ± 1.22	
BCTQs month 1	17.17 ± 0.63	<0.001	18.43 ± 0.93	<0.001
BCTQs month 3	15.76 ± 0.50	<0.001	18.13 ± 1.02	<0.001
BCTQs month 6	14.14 ± 0.45	<0.001	16.20 ± 0.86	<0.001
BCTQf-Pre	19.23 ± 1.08		18.13 ± 0.65	
BCTQf month 1	12.24 ± 0.55	<0.001	14.40 ± 0.70	0.001
BCTQf month 3	10.79 ± 0.40	<0.001	13.63 ± 0.66	<0.001
BCTQf month 6	10.41 ± 0.48	<0.001	12.93 ± 0.65	<0.001
FP-Pre (kg)	3.27 ± 0.28		3.74 ± 0.11	
FP month 1	4.06 ± 0.27	0.002	4.26 ± 0.18	0.071
FP month 3	4.13 ± 0.29	<0.001	4.22 ± 0.17	0.040
FP month 6	4.45 ± 0.23	<0.001	4.68 ± 0.23	0.001
SNCV-Pre (m/s)	30.18 ± 1.29		32.35 ± 1.10	
SNCV month 1	32.45 ± 1.25	<0.001	34.74 ± 1.21	<0.001
SNCV month 3	32.82 ± 1.27	<0.001	35.05 ± 1.28	<0.001
SNCV month 6	33.92 ± 1.34	<0.001	36.17 ± 1.34	<0.001
DML-Pre (ms)	5.66 ± 0.27		5.21 ± 0.23	
DML month 1	5.28 ± 0.23	<0.001	4.96 ± 0.22	0.041
DML month 3	5.26 ± 0.25	0.006	4.98 ± 0.22	0.016
DML month 6	5.18 ± 0.26	0.001	4.74 ± 0.19	<0.001
CSA-Pre (mm^2^)	14.01 ± 0.82		12.91 ± 0.81	
CSA month 1	11.86 ± 0.76	<0.001	11.72 ± 0.81	<0.001
CSA month 3	11.35 ± 0.74	<0.001	11.23 ± 0.72	<0.001
CSA month 6	10.93 ± 0.75	<0.001	10.87 ± 0.76	<0.001

SE = Standard error; VAS = Visual analog scale; BCTQ = Boston Carpal Tunnel Syndrome Questionnaire (s = severity and f = function); FP = Finger pinch; SNCV = Sensory nerve conduction velocity; DML = Distal motor latency; CSA = Cross-sectional area; Pre = Pretreatment.

**Figure 3:**
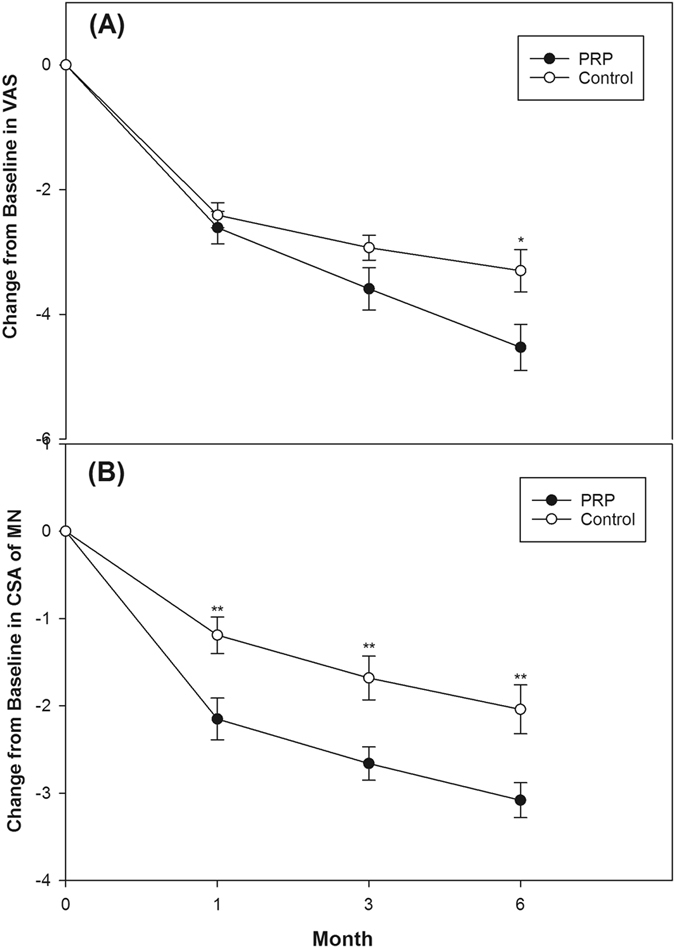
Mean change from baseline in visual analog scale (VAS) and cross-sectional area (CSA) of medain nerve (MN) in both groups (mean ± standard error). (**A**) PRP group had significant improvement of VAS compared with control group at 6^th^ month (p < 0.05). (**B**) PRP group had significant improvement of CSA compared with control group at all follow-up assessments (p < 0.01). (*p < 0.05, **p < 0.01. Independent t-test was used).

**Figure 4:**
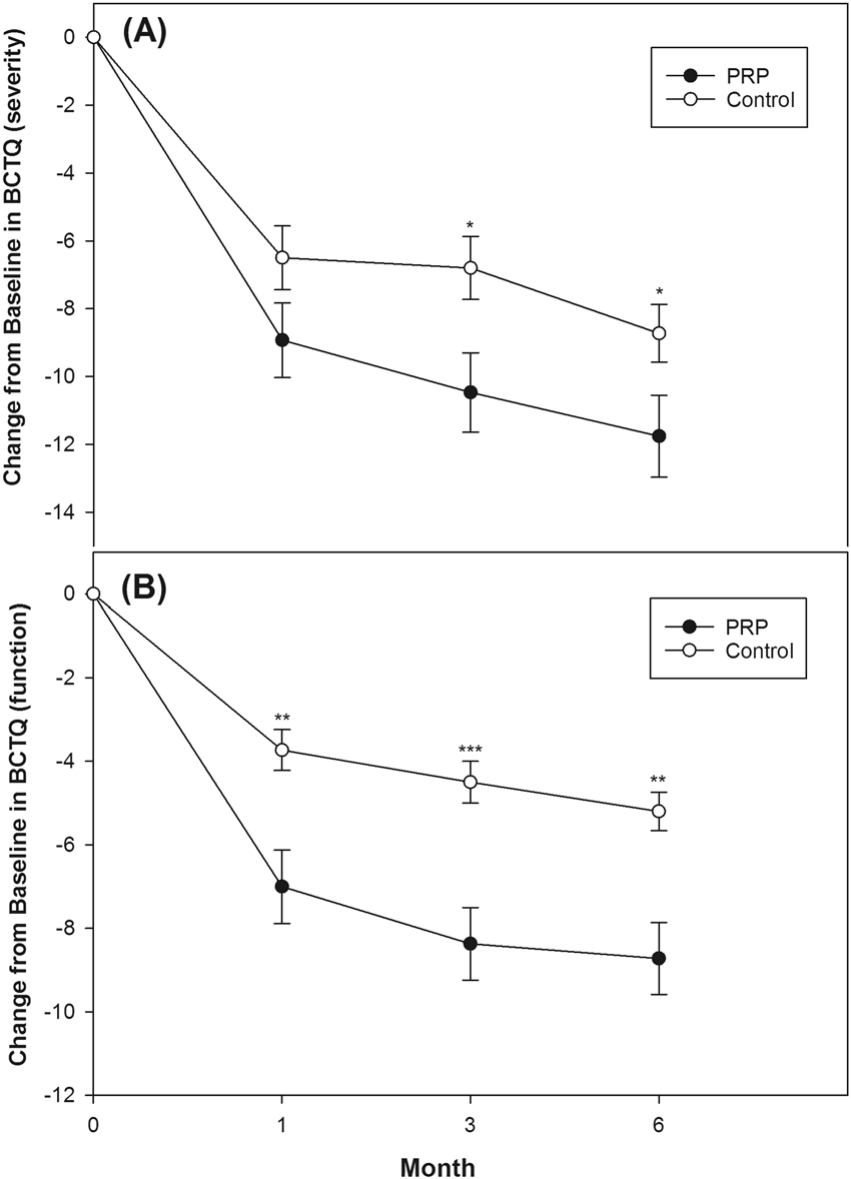
Mean change from baseline in Boston Carpal Tunnel Syndrome Questionnaire (BCTQ) in both groups (mean ± standard error). (**A**) PRP group had significant improvement of BCTQ (severity) compared with control group at 3^rd^ and 6^th^ month (p < 0.05). (**B**) PRP group had significant improvement of BCTQ (function) compared with control group at all follow-up assessments (p < 0.01). (*p < 0.05, **p < 0.01, ***p < 0.001. Independent t-test was used).

Comparing the two groups, there was significantly greater enhancement in the PRP group at all follow-up time points in the VAS scores, BCTQ scores, and CSA of the MN (except for the 1^st^ and 3^rd^ month VAS score and 1^st^ month BCTQ-severity score) and this tendency became more pronounced as the follow-up duration increased (Table 4, Fig. 3 and 4). Although a tendency towards increased FP with a longer follow-up period was found in the PRP group compared with the control group, the difference was not statistically significant (Table 4). The difference in SNCV and DML between the two groups was not statistically significant at all follow-up assessments (Table 4). No side-effects or nerve trauma were observed in either of the two groups. All patients reported taking no additional medicine or receiving other therapies throughout the study.

**Table 4:** Changes of outcome variables from baseline at 1, 3 and 6 month in the PRP group compared with the control group.

	PRP group (n = 30)	Control group (n = 30)	*p* value
	Mean difference ± SE	Mean difference ± SE	
VAS-Pre			
VAS month 1	−2.61 ± 0.26	−2.41 ± 0.20	0.540
VAS month 3	−3.59 ± 0.34	−2.93 ± 0.20	0.104
VAS month 6	−4.53 ± 0.37	−3.30 ± 0.34	0.018
BCTQs-Pre			
BCTQs month 1	−8.93 ± 1.10	−6.50 ± 0.94	0.098
BCTQs month 3	−10.47 ± 1.17	−6.80 ± 0.93	0.017
BCTQs month 6	−11.76 ± 1.21	−8.73 ± 0.85	0.045
BCTQf-Pre			
BCTQf month 1	−7.00 ± 0.88	−3.73 ± 0.49	0.002
BCTQf month 3	−8.37 ± 0.87	−4.50 ± 0.50	<0.001
BCTQf month 6	−8.72 ± 0.86	−5.20 ± 0.46	0.001
FP-Pre (kg)			
FP month 1	0.74 ± 0.16	0.53 ± 0.17	0.384
FP month 3	0.81 ± 0.15	0.49 ± 0.15	0.138
FP month 6	1.12 ± 0.15	0.95 ± 0.20	0.482
SNCV-Pre (m/s)			
SNCV month 1	2.26 ± 0.18	2.39 ± 0.41	0.779
SNCV month 3	2.64 ± 0.40	2.70 ± 0.39	0.917
SNCV month 6	3.74 ± 0.60	3.81 ± 0.54	0.925
DML-Pre (ms)			
DML month 1	−0.38 ± 0.07	−0.25 ± 0.08	0.199
DML month 3	−0.40 ± 0.10	−0.23 ± 0.06	0.157
DML month 6	−0.48 ± 0.10	−0.47 ± 0.08	0.934
CSA-Pre (mm^2^)			
CSA month 1	−2.15 ± 0.24	−1.19 ± 0.21	0.004
CSA month 3	−2.66 ± 0.19	−1.68 ± 0.25	0.003
CSA month 6	−3.08 ± 0.20	−2.04 ± 0.28	0.004

VAS = Visual analog scale; BCTQ = Boston Carpal Tunnel Syndrome Questionnaire (s = severity and f = function); FP = Finger pinch; SNCV = Sensory nerve conduction velocity; DML = Distal motor latency; CSA = Cross-sectional are; Pre = Pretreatment; SE = Standard error.

## Discussion

This is the first prospective, randomized, single-blind controlled trial to explore the efficacy of PRP for patients with mild-to-moderate CTS. We demonstrated that PRP significantly reduced pain severity, ameliorated disability, and improved CSA of MN 6 months post-treatment.

PRP is considered as a safe treatment and practiced in many disciplines of medicine. It is an autologous preparation utilizing the patient’s own blood. Increasing evidence has highlighted the positive effects of PRP on peripheral nerve regeneration with acceptable safety profiles in experimental studies^9–16^. Farrag *et al*.^9^ demonstrated beneficial effects of PRP compared with platelet poor plasma (PPP) for facial nerve regeneration in a rat model. Sariguney *et al*.^10^ showed that PRP enhanced the remyelination of the sciatic nerve in an end-to-end neurorrhaphy rat model. Ding *et al*.^11^ applied PRP to the site of bilateral nerve-crush rat model and the findings revealed a significant effect on cavernous regeneration and functional recovery. Cho *et al*.^12^ reported that PRP could promote facial nerve regeneration in a facial nerve axotomy model. Giannessi *et al*.^13^ commented that PRP could be a nerve guide to diminish scar reaction during axonal regeneration in a rat sciatic nerve model. Another study revealed that PRP could limit nerve damage 12 weeks post-injection in a rabbit model of 10% dextrose-induced MN injury (carpal tunnel model)^14^. Sanchez *et al*.^15^ found that PRP hastened functional axon recovery in an ovine model. Zheng *et al*.^16^ showed that PRP could stimulate Schwann cell proliferation, secretion of nerve growth factor, and neurotrophic factor *in vitro*. In contrast, Piskin *et al*.^38^ reported that PRP does not enhance axonal regeneration of peripheral nerve repair in a rat model.

The clinical benefit of PRP in peripheral neuropathy is an interesting field of research. In 2014, Anjayani *et al*.^17^ first reported a randomized, double-blind, control trial study to prove that a 1-mL PRP perineural injection could improve pain scores using a VAS, and the two-point discrimination test of peripheral neuropathy, in patients with Hansen’s disease compared with a 1-mL PPP injection, 2 weeks after the injection of both types of plasma (n = 30 vs. n = 30, respectively). Subsequently, Sánchez *et al*.^18^ described a patient with recalcitrant peroneal nerve palsy who showed partial recovery and obvious improvement in the electrophysiological study 21 months after the first PRP injection (7 sessions of PRP injection in total). Scala *et al*.^19^ revealed that PRP has a positive effect on, and is protective against, facial nerve neurological deficits in patients undergoing superficial parotidectomy compared with a placebo group in a small randomized control trial (n = 10 vs. n = 10, respectively). In 2015, Malahias *et al*.^20^ first used an ultrasound-guided injection of 1–2 mL of PRP in patients with mild CTS (n = 14, no control group) with positive mid-term outcomes (3 months). Recently, Uzun *et al*.^21^ performed a non-randomized, single-blind trial to compare the effect of PRP with steroid injection in patients with minimal to mild CTS (n = 20 vs. n = 20) by using blind injection. Although they showed that the PRP group had a significant improvement of BCTQ (both symptom and function scores) 3 months post-treatment compared with steroid group, the difference was not significant at the 6 month follow-up. Moreover, there was no significant change between the two groups in the electrophysiological measurements.

Even though positive effects were shown in the above clinical studies, most of these studies enrolled small patient numbers and lacked long-term follow-up. The findings of our study validate the outcomes of the above-mentioned studies. Compared with study of Uzun *et al*.^21^, we recruited more severe CTS (80% moderate vs. minimal to mild CTS, respectively), larger sample size (n = 30 vs. n = 20, respectively), and more precise injection (ultrasound-guided vs. blind injection). Moreover, the effects of PRP in our study persisted for at least 6 months, which was more obvious and lasting than the results from Uzun *et al*.^21^. Our study showed the most positive clinical outcomes using PRP for patients with CTS to date. Furthermore, the tendency for improvement in VAS, BCTQ, and CSA compared with the baseline or control group of our study seemed more pronounced with a longer follow-up duration (Table 4, Fig. 3 and 4). Therefore, we believe the effect could continue for more than 6 months if the follow-up period is extended.

Although significant enhancement of FP, SNCV, and DML compared with pretreatment data was observed in both groups of the present study, the differences between the groups were not significant (Table 4). Moreover, any obvious tendency towards an increased difference between SNCV and DML was not seen; for example, such as FP in the PRP group compared with the control group. Uzun *et al*.^21^ revealed no significant improvement of SNCV and DML during the 6-month follow-up (except third-month SNCV) after PRP injection in patients with minimal to mild CTS in contrast with our findings. Most patients in our study diagnosed with moderate CTS may have had different results compared with those of Uzun’s study. Moreover, different sample sizes and techniques of injection would also produce different results. Our results were compatible with Uzun’s findings in that the improved symptoms between groups did not correlate with the improvement of electrophysiological measurements. The discordance between symptoms and electrophysiological improvement was expected because routine electrophysiological studies examine mainly large myelinated fibers rather than small sensory fibers which may be responsible for some symptoms of CTS^39^. Indeed, the electrodiagnostic measurement has a limited role in predicting the therapeutic outcome for CTS after surgery and conservative treatment in previous studies^23, 40–42^.

The actual mechanism underlying the effects of PRP in neuropathy is unclear. PRP has been reported to contain a variety of growth factors, including platelet-derived growth factor (PDGF), transforming growth factor-β (TGF-β), insulin-like growth factor (IGF), epidermal growth factor, fibroblast growth factor (FGF), keratinocyte growth factor, hepatocyte growth factor (HGF), nerve growth factor (NGF) and vascular endothelial growth factor^43–45^. These growth factors have been suggested to play a positive role in the regeneration of injured peripheral nerves^45–49^. Chen *et al*.^46^ suggested that FGF facilitates angiogenesis and acts as a neurotrophic agent during facial nerve regeneration in the pig model. Oya *et al*.^47^ revealed that PDGF-β is a Schwann cell mitogen and survival factor in the nerve crush-injured rat. Increased PDGF-β level after nerve injury might aid peripheral nerve regeneration. TGF promoted neuron survival^48^ and IGF particularly enhanced the growth of corticospinal motor neurons axons *in vitro* ^49^. Moreover, HGF and NGF have also been shown to enhance axonal sprouting^45^.

Hydrodissection via PRP injection could have contributed to the effect of PRP on CTS in our study. Decreased blood flow to the MN due to progressive compression of the MN is the major mechanism for CTS^50^. Perineural hydrodissection around the entrapped nerve would probably potentiate ischemic damage to a nerve by peeling the MN from surrounding tissues (flexor retinaculum and connective tissue-related compression and adhesion)^51^. Although the effect-duration of hydrodissection on the entrapped nerve is currently unknown^51^, we believe the true effect of one dose of hydrodissection may not significantly persist because the injectate would be absorbed rapidly. Indeed, in our clinical practice, the injectate seems to be clearly absorbed one hour later after injection when rechecked by the ultrasonography. More randomized double-blind trials are necessary to examine the effects of hydrodissection in the future.

Although the mechanism of PRP in CTS in the present study are uncertain and probably multifactorial, we could hypothesize possible causes based on the significantly reduced CSA of the MN and improved electrophysiological studies. First, the PRP could promote angiogenesis, neurogenesis, and regeneration via direct effects on the MN itself based on previous experimental studies^9–16^. Second, PRP could reduce the inflammation and swelling of the flexor tenosynovitis^52^ since we performed perineural injection without intraneural injection to prevent direct nerve trauma. Therefore, the PRP could diffusely encase the MN and surrounding soft tissues. The decreased swelling of the flexor tendon would result in reduction of intracarpal pressure exerted on the MN^23^. Finally, the hydrodissection could also contribute some benefits. A CTS animal model with histological studies is needed to explore and differentiate the above mechanism in the future.

There were some limitations in this study. First, the mechanism of PRP was not evaluated in this study. Second, the effect of hydrodissection and placebo from injection in our study cannot be completely excluded. However, a sham-controlled treatment or an active non-PRP injection in the control group was unfeasible and unacceptable in our country because of the invasive procedure of the PRP preparation and injection. Nonetheless, the lack of a sham-control or placebo effect can hardly explain the marked effect of PRP that lasted throughout the 6 months. Further prospective clinical trials are encouraged with sham-controlled treatment. Finally, more studies with multiple strategies are necessary to optimize the dosage regimen of PRP for optimal effects.

In conclusion, this study shows that ultrasound-guided PRP injection is safe and effective for treating CTS. The efficacy of PRP for CTS seems to be a potentially worthwhile area for further study in peripheral neuropathy.

## References

[1] Werner RA, Andary M (2002). Carpal tunnel syndrome: Pathophysiology and clinical neurophysiology. Clin Neurophysiol.

[2] Karadas O, Tok F, Ulaş UH, Odabaşi Z (2011). The effectiveness of triamcinolone acetonide vs. procaine hydrochloride injection in the management of carpal tunnel syndrome: a double-blind randomized clinical trial. Am J Phys Med Rehabil.

[3] O’Connor, D., Marshall, S. & Massy-Westropp, N. Non-surgical treatment (other than steroid injection) for carpal tunnel syndrome. Cochrane Database Syst Rev. CD003219 (2003). 10.1002/14651858.CD003219 PMC6486195 12535461

[4] Katz JN (1998). Maine carpal tunnel study: Outcomes of operative and nonoperative therapy for carpal tunnel syndrome in a community-based cohort. J Hand Surg Am.

[5] Gerritsen AA (2003). Splinting for carpal tunnel syndrome: Prognostic indicators of success. J Neurol Neurosurg Psychiatry.

[6] Huisstede BM (2010). Carpal tunnel syndrome. Part i: Effectiveness of nonsurgical treatments–a systematic review. Arch Phys Med Rehabil.

[7] Ahcan U, Arnez ZM, Bajrovic F, Zorman P (2002). Surgical technique to reduce scar discomfort after carpal tunnel surgery. J Hand Surg Am.

[8] Sampson S, Gerhardt M, Mandelbaum B (2008). Platelet rich plasma injection grafts for musculoskeletal injuries: A review. Current reviews in musculoskeletal medicine.

[9] Farrag TY, Lehar M, Verhaegen P, Carson KA, Byrne PJ (2007). Effect of platelet rich plasma and fibrin sealant on facial nerve regeneration in a rat model. The Laryngoscope.

[10] Sariguney Y (2008). Effect of platelet-rich plasma on peripheral nerve regeneration. Journal of reconstructive microsurgery.

[11] Ding XG (2009). The effect of platelet-rich plasma on cavernous nerve regeneration in a rat model. Asian journal of andrology.

[12] Cho HH (2010). Effect of neural-induced mesenchymal stem cells and platelet-rich plasma on facial nerve regeneration in an acute nerve injury model. The Laryngoscope.

[13] Giannessi E (2014). An autologously generated platelet-rich plasma suturable membrane may enhance peripheral nerve regeneration after neurorraphy in an acute injury model of sciatic nerve neurotmesis. Journal of reconstructive microsurgery.

[14] Park GY, Kwon DR (2014). Platelet-rich plasma limits the nerve injury caused by 10% dextrose in the rabbit median nerve. Muscle & nerve.

[15] Sanchez, M. *et al*. Ultrasound-guided plasma rich in growth factors injections and scaffolds hasten motor nerve functional recovery in an ovine model of nerve crush injury. J Tissue Eng Regen Med. [Epub ahead of print] (2015). 10.1002/term.2079 26876895

[16] Zheng C (2016). Effect of platelet-rich plasma (PRP) concentration on proliferation, neurotrophic function and migration of Schwann cells *in vitro*. J Tissue Eng Regen Med.

[17] Anjayani S (2014). Sensory improvement of leprosy peripheral neuropathy in patients treated with perineural injection of platelet-rich plasma. International journal of dermatology.

[18] Sanchez M, Yoshioka T, Ortega M, Delgado D, Anitua E (2014). Ultrasound-guided platelet-rich plasma injections for the treatment of common peroneal nerve palsy associated with multiple ligament injuries of the knee. Knee surgery, sports traumatology, arthroscopy: official journal of the ESSKA.

[19] Scala M (2014). The use of platelet-rich plasma gel in patients with mixed tumour undergoing superficial parotidectomy: A randomized study. In vivo (Athens, Greece).

[20] Malahias MA, Johnson EO, Babis GC, Nikolaou VS (2015). Single injection of platelet-rich plasma as a novel treatment of carpal tunnel syndrome. Neural regeneration research.

[21] Uzun H, Bitik O, Uzun Ö, Ersoy US, Aktaş E (2016). Platelet-rich plasma versus corticosteroid injections for carpal tunnel syndrome. J Plast Surg Hand Surg.

[22] Moher D (2010). CONSORT 2010 explanation and elaboration: updated guidelines for reporting parallel group randomised trials. BMJ.

[23] Wu YT (2016). Effect of radial shock wave therapy for carpal tunnel syndrome: A prospective randomized, double-blind, placebo-controlled trial. J Orthop Res.

[24] Jablecki CK (2002). Practice parameter: Electrodiagnostic studies in carpal tunnel syndrome. Report of the american association of electrodiagnostic medicine, american academy of neurology, and the american academy of physical medicine and rehabilitation. Neurology.

[25] You H, Simmons Z, Freivalds A, Kothari MJ, Naidu SH (1999). Relationships between clinical symptom severity scales and nerve conduction measures in carpal tunnel syndrome. Muscle & nerve.

[26] Rossi S (1994). Sensory neural conduction of median nerve from digits and palm stimulation in carpal tunnel syndrome. Electroencephalogr Clin Neurophysiol.

[27] Padua L, LoMonaco M, Valente EM, Tonali PA (1996). A useful electrophysiologic parameter for diagnosis of carpal tunnel syndrome. Muscle & nerve.

[28] Padua L (1997). Neurophysiological classification and sensitivity in 500 carpal tunnel syndrome hands. Acta Neurol Scand.

[29] Napolitano M (2012). Autologous platelet gel for tissue regeneration in degenerative disorders of the knee. Blood transfusion = Trasfusione del sangue.

[30] Wong SM, Griffith JF, Hui AC, Tang A, Wong KS (2002). Discriminatory sonographic criteria for the diagnosis of carpal tunnel syndrome. Arthritis Rheum.

[31] Wu YT (2014). Ultrasound-guided pulsed radiofrequency stimulation of the suprascapular nerve for adhesive capsulitis: A prospective, randomized, controlled trial. Anesth Analg.

[32] Lee JY, Park Y, Park KD, Lee JK, Lim OK (2014). Effectiveness of ultrasound-guided carpal tunnel injection using in-plane ulnar approach: A prospective, randomized, single-blinded study. Medicine.

[33] Huskisson EC (1974). Measurement of pain. Lancet.

[34] Levine DW (1993). A self-administered questionnaire for the assessment of severity of symptoms and functional status in carpal tunnel syndrome. J Bone Joint Surg Am.

[35] Jablecki CK, Andary MT, So YT, Wilkins DE, Williams FH (1993). Literature review of the usefulness of nerve conduction studies and electromyography for the evaluation of patients with carpal tunnel syndrome. Aaem quality assurance committee. Muscle & nerve.

[36] Stegink Jansen CW, Simper VK, Stuart HG, Pinkerton HM (2003). Measurement of maximum voluntary pinch strength: Effects of forearm position and outcome score. J Hand Ther.

[37] Faul F, Erdfelder E, Lang AG, Buchner A (2007). G* power 3: A flexible statistical power analysis program for the social, behavioral, and biomedical sciences. Behavior research methods.

[38] Piskin A (2009). Platelet gel does not improve peripheral nerve regeneration: an electrophysiological, stereological, and electron microscopic study. Microsurgery.

[39] Soyupek F (2012). Determining the effectiveness of various treatment modalities in carpal tunnel syndrome by ultrasonography and comparing ultrasonographic findings with other outcomes. Rheumatol Int.

[40] Unglaub F, Wolf E, Goldbach C, Hahn P, Kroeber MW (2008). Subjective and functional outcome after revision surgery in carpal tunnel syndrome. Arch Orthop Trauma Surg.

[41] Manente G (2001). An innovative hand brace for carpal tunnel syndrome: a randomized controlled trial. Muscle Nerve.

[42] Chen LC (2015). Ultrasound-Guided Pulsed Radiofrequency for Carpal Tunnel Syndrome: A Single-Blinded Randomized Controlled Study. PLoS One.

[43] Eppley BL, Woodell JE, Higgins J (2004). Platelet quantification and growth factor analysis from platelet-rich plasma: Implications for wound healing. Plastic and reconstructive surgery.

[44] Nikolidakis D, Jansen JA (2008). The biology of platelet-rich plasma and its application in oral surgery: Literature review. Tissue engineering. Part B, Reviews.

[45] Sanchez M (2016). Platelet-rich plasma, a source of autologous growth factors and biomimetic scaffold for peripheral nerve regeneration. Expert Opin Biol Ther.

[46] Chen YS (1999). Effects of basic fibroblast growth factor (bfgf)-neutralizing antibody and platelet factor 4 on facial nerve regeneration. Experimental neurology.

[47] Oya T (2002). Platelet-derived growth factor-b expression induced after rat peripheral nerve injuries. Glia.

[48] McTigue DM, Popovich PG, Morgan TE, Stokes BT (2000). Localization of transforming growth factor-beta1 and receptor mrna after experimental spinal cord injury. Experimental neurology.

[49] Ozdinler PH, Macklis JD (2006). Igf-i specifically enhances axon outgrowth of corticospinal motor neurons. Nature neuroscience.

[50] Evans KD, Roll SC, Volz KR, Freimer M (2012). Relationship between intraneural vascular flow measured with sonography and carpal tunnel syndrome diagnosis based on electrodiagnostic testing. J Ultrasound Med.

[51] Cass SP (2016). Ultrasound-Guided Nerve Hydrodissection: What is it? A Review of the Literature. Curr Sports Med Rep.

[52] Takamura, M., Yasuda, T., Nakano, A., Shima, H. & Neo, M. The effect of platelet-rich plasma on Achilles tendon healing in a rabbit model. Acta Orthop Traumatol Turc. [Epub ahead of print] (2016). 10.1016/j.aott.2016.12.001 PMC6197299 28027872

